# Guillermo Rivera: *Cuídate de la justicia Boliviana*

**DOI:** 10.1192/bjb.2024.51

**Published:** 2024-10

**Authors:** Claire Mckenna

**Figure:**
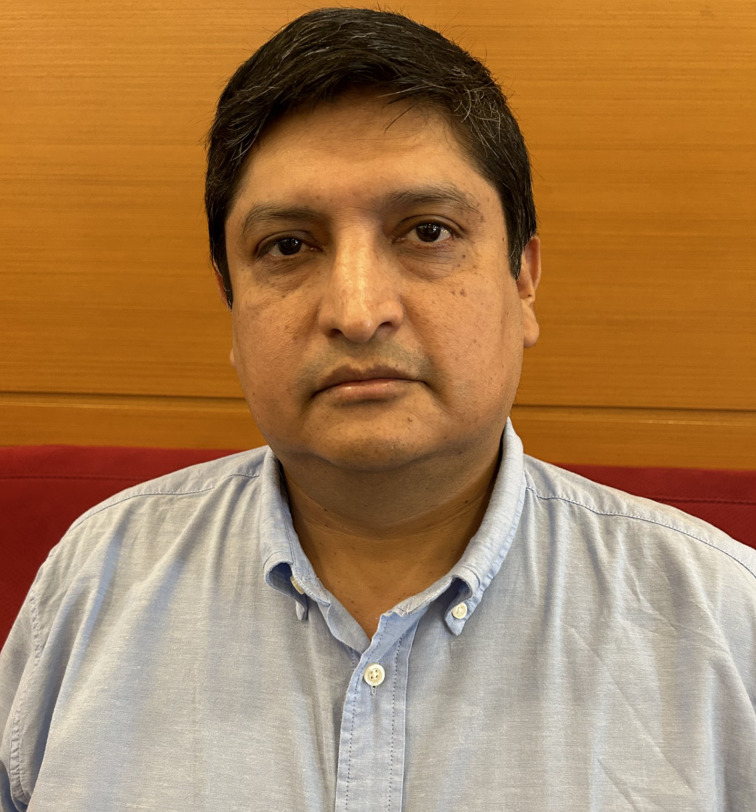

In the Western world, our interest in prison systems in South America often carries a voyeuristic tinge, exemplified by shows such as the TV docuseries ‘Inside the world's toughest prisons’, on Netflix. Professor Guillermo Rivera cautions us to ‘be mindful of the potential for reinforcing stereotypes’. These programmes don't give a picture of the efforts being made for reform, he says. He allows, however, that there are kernels of truth in such depictions: ‘Prison conditions [in Bolivia] can be dangerous for both prisoners and guards, with overcrowding contributing to tensions and conflicts. The presence of self-governance systems within some prisons, where inmates play a role in maintaining order, can influence safety’. As of 2020, whole families no longer live in prisons but children up to the age of 6 are still allowed to stay with their incarcerated mothers.

Sensationalist portrayals of Bolivian prisons also risk losing the humanity of those incarcerated. In Boxes 1 and 2 Professor Rivera tells us the story of two anonymised patients, who represent common threads of experience for prisoners he has worked with.

Box 1 Jorge

‘Jorge’ is a 38-year-old man diagnosed with schizophrenia who was accused of a violent robbery at the age of 29, although the case was never proven. Currently, Jorge resides in prison under precarious conditions and is unable to afford medication because of financial constraints. His family has abandoned him, and attempts by the psychiatrist to contact them have been rejected. Without legal representation, he seems to be forgotten by the system, living in the corridors of the prison.

Box 2 Luis

‘Luis’ is a Bolivian man who lived outside South America until early adulthood. He was diagnosed with delusional disorder as an adolescent. In early adulthood, he returned to Bolivia. He discontinued his medication because relatives believed he no longer needed it. Subsequently, he developed paranoid delusions. Luis fatally stabbed a close family member, believing they had been replaced by a demonic entity. After the murder, he waited for the police and was sent to prison. Wealthy relatives from abroad hired a lawyer, and a psychiatrist was asked to assess the patient and confirm the diagnosis. At that time, he was not receiving medication.

After the forensic evaluation, the judge ordered Luis to be interned in a psychiatric facility. It has been several years since that order, but the opposing party has objected and hindered the judicial order. Luis remains in prison, where he is receiving medication administered by another inmate, who voluntarily assists the psychiatrist in distributing the medication. Luis is currently studying for a degree, receives daily visits and is in a romantic relationship.

These days, Rivera tries to drive change mainly through his work as an academic, with a small clinical practice. His primary research interests focus on the mental health of prison populations, mental health service development and early intervention in psychosis. He has recently become the first psychiatrist to hold the position of ‘full academician’ at the National Academy of Sciences of Bolivia. He also takes great pride in his role as the founder and editor of the *Revista Boliviana de Psiquiatría* [*Bolivian Journal of Psychiatry*].

The challenges of improving mental healthcare in a country with 105 registered psychiatrists serving a population of 11 million cannot be overestimated. The biggest barrier for the population, however, is poverty. ‘The primary factor contributing to inequality in accessing mental health services in general in Bolivia is economic income,’ Rivera explains. He continues ‘This is because the majority of mental health services are fee-based. The rural population entirely lacks such services and 90% of the Bolivian prison population lacks access to any psychiatric services’. Rivera told me that if you don't have money, it's extremely difficult as a mentally disordered offender to obtain either a lawyer or a psychiatrist to help you get out of prison. There are 90 prisons in Bolivia and only one (the biggest prison, ‘Palmasola’ in Santa Cruz) has a state-funded psychiatrist.

Political instability and corruption within the justice system and policing are another major challenge to reform.^1^ Rivera's concerns about his own personal safety influence what opinions he can express about that in this interview. He quotes a well-known Latin American proverb, which after some ‘politically incorrect’ exhortations in relation to other South American countries, ends with the warning: ‘*Cuídate de la justicia Boliviana* [Beware Bolivian justice]’.

This interview has been edited and condensed for clarity.

**What are conditions like in prisons in Bolivia? Are there any aspects that would surprise European observers?**

Some Bolivian prisons have unique systems of self-governance, where inmates play a significant role in the day-to-day operations of the facilities. This can include elected leaders among the inmate population, who help maintain order and negotiate with prison authorities.

In Bolivia, there is a practice where prisoners are required to pay for the rent of their cells to inmates who own them. The more one pays for rent, the more comfortable the cell is expected to be. Unfortunately, those who cannot afford to pay may find themselves relegated to sleeping in the prison hallways. Prisoners with no money may have to wash other prisoners’ clothes or clean their rooms to get money for food. This system adds a layer of economic disparity within the prison environment, impacting the living conditions and space available to inmates based on their financial means.

These self-governance systems within prisons are often born out of necessity, especially in the context of overcrowded and resource-constrained facilities. While inmates may have a role in certain aspects of prison life, the ultimate authority rests with the prison administration and relevant authorities. The coexistence of these informal structures with the formal prison hierarchy can vary between different prisons in Bolivia.

In many cases, prisons lack adequate resources, training or staffing levels, impacting the ability of guards to maintain order and ensure security.

**Are people able to have their basic needs met, for example for food and beds? What does it take to survive?**

Surviving in Bolivian prisons can be challenging, and individuals may need to navigate complex social dynamics, informal power structures among inmates and sometimes limited resources. The ability to have basic needs met may depend on various factors, including an individual's financial means, relationships with other inmates and the specific conditions of the prison.

According to the Institute for Therapy and Research on the Aftermath of Torture and State Violence (ITEI), Bolivian penitentiary centres have an overpopulation rate of 269% – one of the highest figures in the region.

**Is there much behavioural disturbance in Bolivian prisons and how is it managed?**

The formation of power groups within Bolivian penitentiary facilities, vying for dominance and control, can contribute to behavioural disturbances. These power dynamics may lead to conflicts, violence and challenges in maintaining order within the prisons. Riots in Bolivian prisons are often suppressed with extreme violence, and prison deaths are frequent.

**What is the most common offence for which prisoners are incarcerated?**

Drug-related offences contribute significantly to prison populations.

**What percentage of prisoners are ‘on remand’ awaiting trial?**

Approximately 70% of prisoners are in pretrial detention. These statistics are derived from the 2019 Prison Census, conducted using data from the Supreme Court of Justice, the Police, the Service for the Prevention of Torture, and the Penitentiary System.

**What happens to defendants with mental health problems in Bolivia who might be found ‘unfit to plead’ or ‘not guilty by reason of insanity’ in the UK?**

In Bolivia, when a defendant is found to have mental health problems and needs psychiatric evaluation or treatment, the usual procedure involves referring them to a psychiatric hospital. However, the process of this referral can be prolonged, potentially taking years, owing to the slow pace of the judicial system. The delays may be attributed to various factors, including bureaucratic processes, limited resources and the overall inefficiency of the legal system. These delays can have significant implications for the timely access to mental healthcare for individuals within the criminal justice system.

**What happens to prisoners with serious mental health problems or addiction problems?**

In many cases, prisoners with serious mental health problems or addiction issues, particularly in prisons outside urban areas, often find themselves without adequate treatment. They may be confined to their cells or left to wander the prison without receiving the necessary mental health or addiction care. The challenges in providing comprehensive mental health and addiction services in such settings, combined with resource limitations, contribute to a situation where individuals with these problems may not receive the appropriate support and treatment they require.

**What is the prevalence of drug use in prisons in Bolivia and what impact does this have on the incidence and treatment of mental illness?**

Drug use within prison environments is a common issue in Bolivia. There are no studies describing the prevalence of drug use in Bolivian prisons. However, it is generally observed that the use of ‘pasta base’ cocaine is common, especially among individuals with very low incomes. Many of the prisoners who live in corridors are addicts and the other prisoners may pay them [for services such as cleaning] with drugs.

**Is there mental health legislation in Bolivia? What are human rights protections like for people with mental disorders? What are the plans to fill the gaps?**

As of now, Bolivia does not have a mental health law in place. However, there is an ongoing effort towards the development of mental health legislation. Currently, we are engaged in the dissemination of a draft bill crafted by the Bolivian Society of Psychiatry, with support from other mental health professionals, service users, their families and non-governmental organisations [NGOs]. This collaborative initiative reflects a concerted effort to address mental health concerns and establish a legal framework to protect the rights and well-being of individuals dealing with mental health issues in Bolivia.

**Are there any moves to deinstitutionalisation for people with mental illness in Bolivia?**

The proposed mental health law in Bolivia adopts a community-based approach, emphasising the importance of providing mental health services within the community. Unlike many countries in the region, Bolivia has historically experienced significant neglect regarding mental illness. Psychiatric beds are scarce, resulting in a situation where the majority of individuals affected by mental disorders often go without any form of treatment. This underscores the pressing need to substantially enhance access to mental health services in the country and more effectively address the needs of those grappling with mental health challenges.

**In your opinion, what is the association between the trauma history of prisoners and the incidence of mental illness?**

In my experience, many prisoners have a history of adverse life experiences and trauma. For example, studies conducted on incarcerated women, particularly those convicted of murder, revealed a high prevalence of complex post-traumatic stress disorder. This suggests that exposure to traumatic events significantly contributes to the development of mental health challenges among individuals in the prison population.

**Is forensic psychiatry a recognised specialty in Bolivia?**

Yes, it is among the medical societies. There is an increasing interest among local psychiatrists in this field but they still lack institutional recognition from the state.

**What is needed to develop forensic mental health services in general in Bolivia?**

Often, practice between forensic psychologists and psychiatrists is dispersed, lacking a well-established and coordinated team structure. Developing forensic mental health services in Bolivia requires specialised training, collaboration between mental health and legal professionals, and the establishment of dedicated teams.

As I mentioned earlier, among Bolivia's 90 prisons, only one has a state-funded psychiatrist. And that psychiatrist does not have any staff with him. He's alone. He has no psychologists working with him because psychologists are only doing administrative work. There are no nurses. The only people who collaborate with him are other prisoners. Prisoners who work for religious NGOs, who are volunteers. They are used to distribute medication to people with severe mental illness.

In the majority of prisons, there is either no mental health personnel or psychiatrists work in economically precarious conditions.

**If you had to pick one single reform that would most improve healthcare for mentally disordered offenders, what would it be?**

We need to develop a legal framework to protect prisoners with mental disorders and to support the work of the mental health professionals.

**Is there anything that we in the UK could learn from Bolivia with respect to the development of mental health services? I am wondering if perhaps there is a stronger sense of family and/or community in Bolivia and if you think that is something that more atomised societies such as the UK could learn from?**

Bolivia has a strong tradition of community involvement in mental healthcare. This includes the use of traditional healers and community health workers to provide care and support to people with mental illness. This approach recognises the importance of family and community support in the recovery process and could be a valuable lesson for other countries to learn from.

**It sounds like conditions for delivering mental healthcare in Bolivia can be challenging. What keeps you going?**

Indeed, providing mental healthcare in Bolivia comes with its set of challenges, including limited resources and awareness. What keeps me going is the genuine belief in the transformative power of mental health support. Witnessing the positive impact on individuals’ lives, despite the challenges, is immensely rewarding.

Moreover, being a pioneer in advancing mental health awareness in Bolivia adds a sense of purpose to my work. There's a fulfilment in breaking down barriers and fostering understanding within the community. The continuous drive to improve access to mental health services and contribute to a broader societal understanding of mental well-being keeps me motivated and dedicated to my role as a psychiatrist in Bolivia.
